# Supplementary material for: How Reversible Are the Effects of Fumed Silica on Macrophages? A Proteomics-Informed View
Source: Nanomaterials (Basel). 2020 Sep 29;10(10):1939. doi: 10.3390/nano10101939 (PMC7600894; doi:10.3390/nano10101939)
Supplement: Supplementary file 1 [file nanomaterials-10-01939-s001.zip › nanomaterials-919049-si-proof done.pdf]

nanomaterials

Article

How Reversible Are the Effects of Fumed Silica on Macrophages? A Proteomics-Informed View

Anaelle Torres [1], Bastien Dalzon [1], Véronique Collin-Faure [1], Hélène Diemer [2], Daphna Fenel [3], Guy Schoehn [3], Sarah Cianférani [2], Marie Carrière [4] and Thierry Rabilloud [1,*]

[1] Laboratory of Chemistry and Biology of Metals, University Grenoble Alpes, CNRS, CEA, UMR 5249, CEDEX 09, 38054 Grenoble, France; anaelle.torres@cea.fr (A.T.); bastien.dalzon@cea.fr (B.D.); veronique.collin@cea.fr (V.C.-F.)
[2] Laboratoire de Spectrométrie de Masse BioOrganique, Université de Strasbourg, CNRS, IPHC UMR 7178, F-67087 Strasbourg, France; hdiemer@unistra.fr (H.D.); sarah.cianferani@unistra.fr (S.C.)
[3] Institute for Structural Biology, Université Grenoble Alpes, CNRS, CEA, F-38000 Grenoble, France; daphna.fenel@ibs.fr (D.F.); guy.schoehn@ibs.fr (G.S.)
[4] Chimie Interface Biologie pour l'Environnement, la Santé et la Toxicologie (CIBEST), University Grenoble-Alpes, CEA, CNRS UMR 5819, IRIG-SyMMES, F-38054 Grenoble, France; marie.carriere@cea.fr
* Correspondence: thierry.rabilloud@cnrs.fr

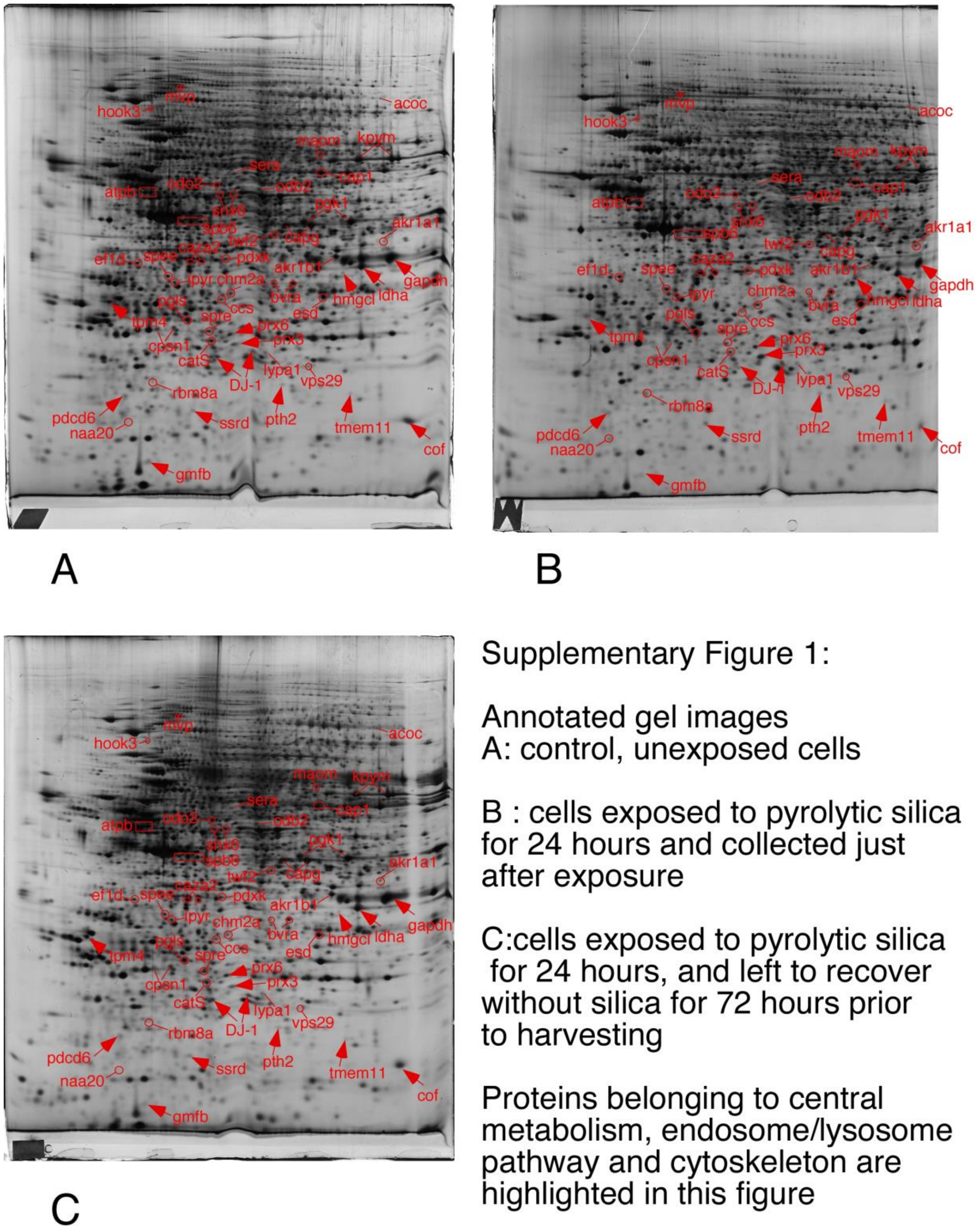
A
B
C
hook3 mvp acoc maom kpym sera cap1 atpb odo2 odb2 snx6 pgk1 spb6 akr1a1 twf2 capg caza2 ef1d spee pdxk akr1b1 gapdh ipyr chm2a bvra hmgcl ldha pgls ccs esd tpm4 spre prx6 prx3 cpsn1 catS lypa1 vps29 DJ-1 rbm8a pth2 pdcd6 naa20 ssrd tmem11 cof gmfb
Supplementary Figure 1:
Annotated gel images
A: control, unexposed cells
B : cells exposed to pyrolytic silica for 24 hours and collected just after exposure
C:cells exposed to pyrolytic silica for 24 hours, and left to recover without silica for 72 hours prior to harvesting
Proteins belonging to central metabolism, endosome/lysosome pathway and cytoskeleton are highlighted in this figure

Figure S1

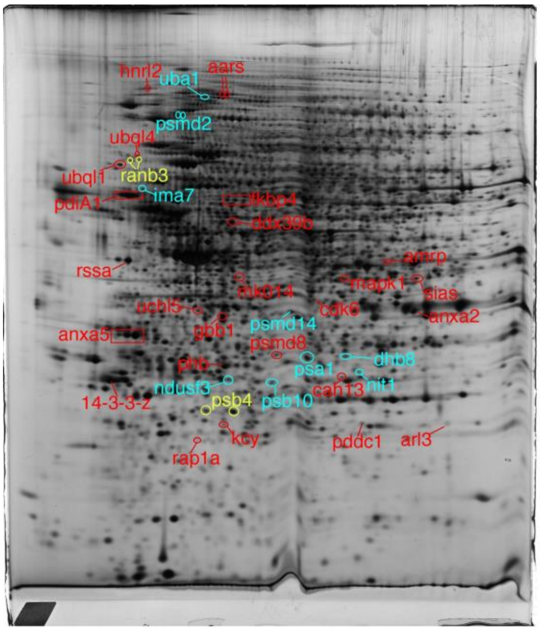

A

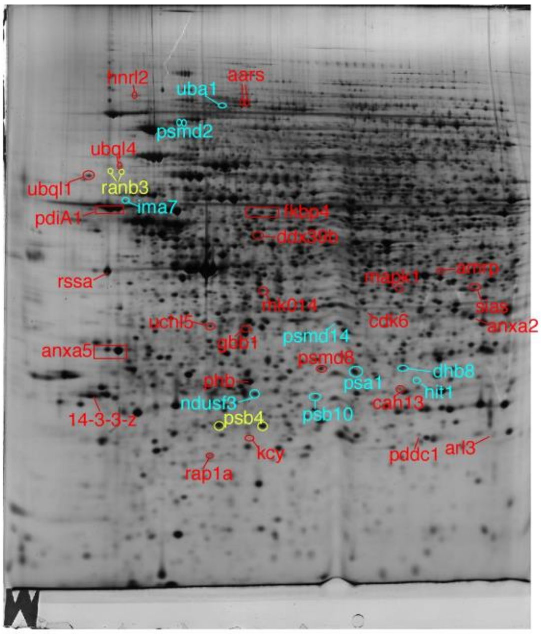

B

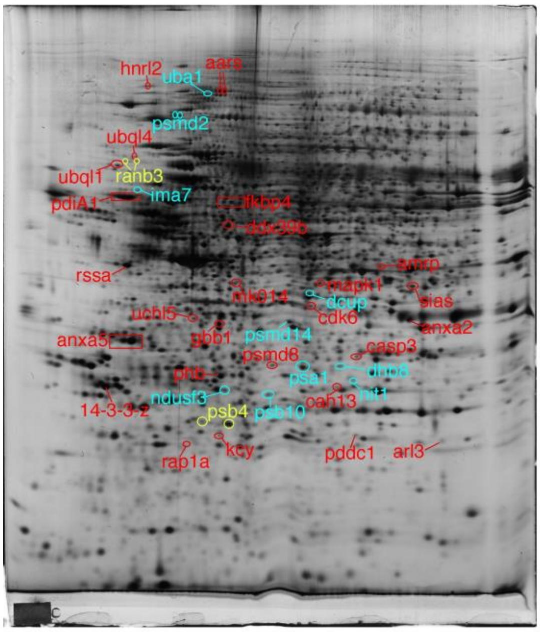

C

Supplementary Figure 2:

Annotated gel images
A: control, unexposed cells

B : cells exposed to pyrolytic silica for 24 hours and collected just after exposure

C:cells exposed to pyrolytic silica for 24 hours, and left to recover without silica for 72 hours prior to harvesting

Proteins belonging to the ubliquitin/ proteasome system, lipid metabolism, signalling, mitochondrion and protein production and folding are highlighted in this figure

Figure S2

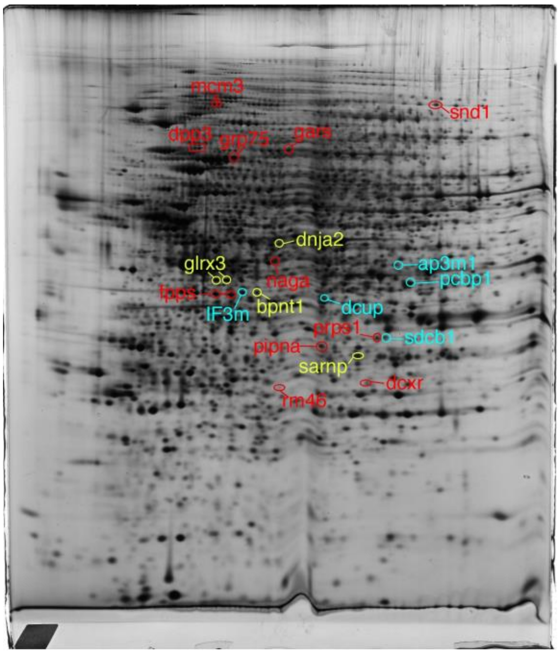

A

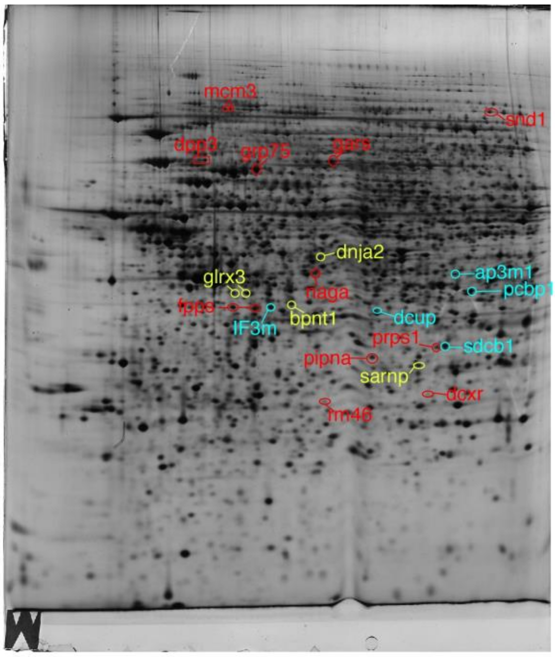

B

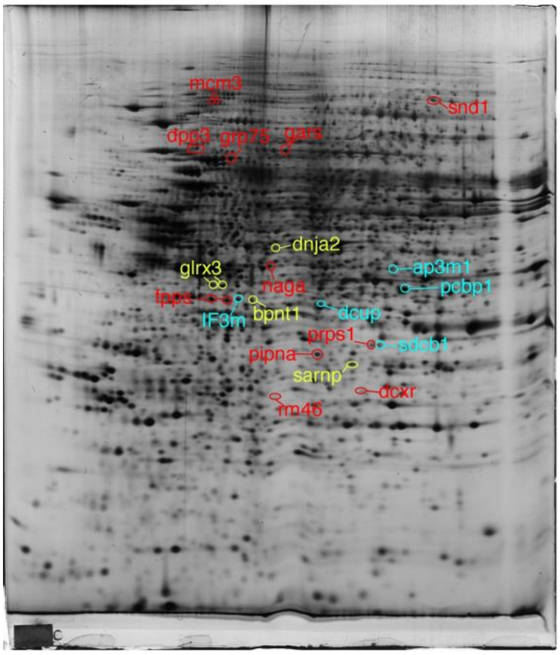

C

Supplementary Figure 3:

Annotated gel images
A: control, unexposed cells

B : cells exposed to pyrolytic silica for 24 hours and collected just after exposure

C:cells exposed to pyrolytic silica for 24 hours, and left to recover without silica for 72 hours prior to harvesting

Otherwise unclassified proteins are highlighted in this figure

Figure S2
