# Supplementary material for: How Reversible Are the Effects of Fumed Silica on Macrophages? A Proteomics-Informed View
Source: Nanomaterials (Basel). 2020 Sep 29;10(10):1939. doi: 10.3390/nano10101939 (PMC7600894; doi:10.3390/nano10101939)
Supplement: Supplementary file 1 [file nanomaterials-10-01939-s001.zip › Table S1.pdf]

Supplementary Table 1: proteins showing a significant change in abundance vs control in one of the two tested conditions

bold: proteins with a change greater in aplitude at the end of the recovery period compared to the change observed immediately after exposure
italics : proteins showing a significant change by the Mann-Whitney U test but not by the Student T test
when present : ac=acidic form; bas=basic form : med=median form

code	name	accession	peptide count	percent seq coverage	ratio acute/ctrl	T test acute vs ctrl	ratio recov/ctrl	T test recov vs ctrl
14-3-3 zeta	14-3-3 protein zeta/delta	P63101	21	75%	0,76290	0,02651	0,99007	0,92966
aars ac	Alanine--tRNA ligase, cytoplasmic	Q8BGQ7	39	49%	1,57024	0,01813	1,18233	0,59140
aars bas	Alanine--tRNA ligase, cytoplasmic	Q8BGQ7	46	55%	1,49569	0,03230	1,07151	0,81469
aars ∑	Alanine--tRNA ligase, cytoplasmic	Q8BGQ7			1,53022	0,02138	1,12284	0,70052
acoc	Cytoplasmic aconitate hydratase	P28271	5	5%	0,50617	0,04263	1,03364	0,90416
akr1a1	***Alcohol dehydrogenase [NADP(+)]***	***Q9JII6***	***17***	***66%***	***1,23212***	***0,38746***	***0,75380***	***0,07708***
**akr1b1**	**Aldose reductase**	**P45376**	**15**	**50%**	**0,90708**	**0,33590**	**0,82794**	**0,03855**
amrp	Alpha-2-macroglobulin receptor-associated protein	P55302	29	61%	0,61065	0,00437	0,91219	0,62467
anxa2	Annexin A2	P07356	27	66%	0,64136	0,01630	0,97415	0,76721
anxa5 ac	Annexin A5	P48036	19	62%	0,50928	0,04541	0,65936	0,12303
anxa5 bas	Annexin A5	P48036	29	83%	1,13620	0,04198	1,14672	0,09548
anxa5 med	Annexin A5	P48036	23	56%	0,81264	0,26440	0,94032	0,62948
anxa5 ∑	Annexin A5	P48036			0,86677	0,22537	0,95345	0,47953
ap3m1	AP-3 complex subunit mu-1	Q9JKC8	18	67%	2,23364	0,01103	0,79387	0,64513
arl3	ADP-ribosylation factor-like protein 3	Q9WUL7	11	69%	2,17187	0,01683	1,22052	0,43727
atpb ac	ATP synthase subunit beta, mitochondrial	P56480	16	39%	1,46182	0,04489	1,22323	0,32400
atpb bas	ATP synthase subunit beta, mitochondrial	P56480	9	27%	1,61813	0,01289	1,21226	0,28437
atpb ∑	ATP synthase subunit beta, mitochondrial	P56480			1,54692	0,01159	1,21726	0,27273
bpnt1	3'(2'),5'-bisphosphate nucleotidase 1	Q9Z0S1	12	45%	2,08832	0,01616	1,18476	0,36510
bvra ac	Biliverdin reductase A	Q9CY64	13	51%	0,40829	0,03776	1,00416	0,98931
bvra bas	*Biliverdin reductase A*	*Q9CY64*	*14*	*59%*	*0,85976*	*0,06744*	*0,92202*	*0,73954*
bvra ∑	Biliverdin reductase A	Q9CY64			0,70094	0,02176	0,95091	0,84054
cah13	Carbonic anhydrase 13	Q9D6N1	4	12%	0,68610	0,04922	0,91080	0,60251

cap1	Adenylyl cyclase-associated protein 1	P40124	9	22%	0,68705	0,03278	1,00948	0,96051
capg	Macrophage-capping protein	P24452	11	34%	0,80400	0,04840	0,91501	0,43314
capg ac	Macrophage-capping protein	P24452	12	49%	0,84106	0,06848	0,88736	0,30541
capg ∑	Macrophage-capping protein	P24452			0,82039	0,04019	0,90278	0,31255
**casp3**	**Caspase-3**	**P70677**	**12**	**53%**	**0,99746**	**0,98635**	**0,68063**	**0,03546**
catS	Cathepsin S	O70370	6	22%	0,71356	0,02330	0,91490	0,32559
caza2 ac	F-actin-capping protein subunit alpha-2	P47754	11	59%	1,79361	0,01906	1,11616	0,23413
caza2 bas	F-actin-capping protein subunit alpha-2	P47754	6	29%	1,02448	0,85172	0,86522	0,25628
caza2 ∑	F-actin-capping protein subunit alpha-2	P47754			1,31082	0,02220	0,95864	0,55954
**ccs**	**Copper chaperone for superoxide dismutase**	**Q9WU84**	**2**	**8%**	**0,94578**	**0,35003**	**1,20553**	**0,03962**
cdk6	Cyclin-dependent kinase 6	Q64261	3	11%	0,69009	0,03320	0,81637	0,31945
**chm2a**	**Charged multivesicular body protein 2a**	**Q9DB34**	**6**	**26%**	**0,94643**	**0,39662**	**0,65042**	**0,00898**
cof bas	*Cofilin-1*	*P18760*	*5*	*42%*	*0,51738*	*0,05847*	*0,94789*	*0,88027*
cpsn1 ac	Calpain small subunit 1	O88456	5	33%	0,55295	0,03978	1,16217	0,26487
cpsn1 bas	Calpain small subunit 1	O88456	9	68%	1,09112	0,71534	0,95775	0,86606
cpsn1 ∑	Calpain small subunit 1	O88456			0,82100	0,11007	1,06036	0,54005
dcup	Uroporphyrinogen decarboxylase	P70697	4	14,00%	0,46458	0,02995	0,89999	0,45250
dcxr	L-xylulose reductase	Q91X52	8	42%	0,30716	0,04586	0,99983	0,99949
ddx39b	Spliceosome RNA helicase Ddx39b	Q9Z1N5	16	46%	1,33840	0,04630	0,99179	0,96254
dhb8	Estradiol 17-beta-dehydrogenase 8	P50171	8	49%	1,43632	0,00789	0,77165	0,19337
dj1 ac	*Protein deglycase DJ-1*	*Q99LX0*	*9*	*69%*	*0,60700*	*0,15160*	*1,00820*	*0,96792*
dj1 bas	*Protein deglycase DJ-1*	*Q99LX0*	*11*	*83%*	*1,64818*	*0,09085*	*0,92780*	*0,77092*
dj1 ∑	Protein deglycase DJ-1	Q99LX0			1,11572	0,45086	0,96892	0,84485
**dnja2**	**DnaJ homolog subfamily A member 2**	**Q9QYJ0**	**4**	**9%**	**0,95942**	**0,68473**	**0,69231**	**0,02057**
dpp3 ac	*Dipeptidyl peptidase 3*	*Q99KK7*	*11*	*22%*	*1,45965*	*0,09187*	*1,35807*	*0,37938*
dpp3 bas	Dipeptidyl peptidase 3	Q99KK7	9	19%	1,76586	0,01153	1,37127	0,34811
dpp3 ∑	Dipeptidyl peptidase 3	Q99KK7			1,63796	0,01459	1,36576	0,35615
ef1d	Elongation factor 1-delta	P57776	5	21%	1,40944	0,00230	1,07337	0,39758
esd	S-formylglutathione hydrolase	Q9R0P3	14	73%	1,25939	0,02840	0,85967	0,09616
fkbp4 ac	Peptidyl-prolyl cis-trans isomerase FKBP4	P30416	13	27,00%	1,17122	0,38770	0,85906	0,46634
fkbp4 bas	Peptidyl-prolyl cis-trans isomerase FKBP4	P30416	23	50%	1,52073	0,18928	0,99570	0,98926

fkbp4 med	*Peptidyl-prolyl cis-trans isomerase FKBP4*	*P30416*	*20*	*43%*	*1,56506*	*0,10775*	*0,90989*	*0,72179*
fkbp4 ∑	Peptidyl-prolyl cis-trans isomerase FKBP4	P30416			1,44136	0,16404	0,92684	0,76864
fpps ac	Farnesyl pyrophosphate synthase	Q920E5	17	63%	1,02992	0,75642	1,13639	0,22162
fpps bas	Farnesyl pyrophosphate synthase	Q920E5	10	31%	1,40391	0,04292	1,24951	0,35046
fpps ∑	Farnesyl pyrophosphate synthase	Q920E5			1,14425	0,12395	1,17097	0,27736
gapdh	Glyceraldehyde-3-phosphate dehydrogenase	P16858	22	59%	0,56124	0,01185	1,02877	0,84796
**gars**	**Glycine--tRNA ligase**	**Q9CZD3**	**22**	**36%**	**1,11067**	**0,72866**	**0,58764**	**0,01867**
gbb1	Guanine nucleotide-binding protein G(I)/G(S)/G(T) subunit beta-1	P62874	7	24%	1,89777	0,01888	0,92893	0,41875
glrx3 ac	Glutaredoxin-3	Q9CQM9	2	9%	1,23829	0,17960	0,97262	0,66012
glrx3 bas	Glutaredoxin-3	Q9CQM9	11	41%	1,45099	0,04340	0,98694	0,88249
glrx3 ∑	Glutaredoxin-3	Q9CQM9			1,35547	0,07363	0,98051	0,79051
gmfb	Glia maturation factor beta	Q9CQI3	8	44%	0,75495	0,02699	1,22229	0,29340
grp75	Stress-70 protein, mitochondrial	O35501	25	43%	1,93665	0,00736	1,02408	0,88684
hmgcl	***Hydroxymethylglutaryl-CoA lyase, mitochondrial***	***P38060***	***7***	***34%***	***1,60154***	***0,12343***	***0,52947***	***0,07353***
hnrl2	Heterogeneous nuclear ribonucleoprotein U-like protein 2	Q00PI9	13	19,00%	0,58894	0,01850	1,07315	0,88827
**hook3**	**Protein Hook homolog 3**	**Q8BUK6**	**4**	**5%**	**1,07412**	**0,65045**	**0,45019**	**0,00519**
iF3M	Eukaryotic translation initiation factor 3 subunit M	Q99JX4	20	64%	1,49603	0,02779	1,03209	0,81144
ima7	Importin subunit alpha-7	O35345	18	41%	2,11765	0,01582	0,84029	0,61907
**imdh2**	**Inosine-5'-monophosphate dehydrogenase 2**	**P24547**	**11**	**21%**	**1,30452**	**0,04136**	**1,33870**	**0,30872**
ipyr	Inorganic pyrophosphatase	Q9D819	5	14%	1,89687	0,02540	0,98832	0,93752
kcy	UMP-CMP kinase	Q9DBP5	10	59%	0,42646	0,03794	1,06299	0,80275
kpym ac	Pyruvate kinase PKM	P52480	19	42%	0,65913	0,04843	0,98415	0,88103
kpym bas	*Pyruvate kinase PKM*	*P52480*	*27*	*61%*	*0,72140*	*0,05389*	*0,78313*	*0,10077*
kpym ∑	Pyruvate kinase PKM	P52480			0,68433	0,04725	0,90280	0,37449
ldha	L-lactate dehydrogenase A chain	P06151	20	44%	0,60178	0,00675	0,91651	0,63780
lypa1	Acyl-protein thioesterase 1	P97823	4	20%	1,40416	0,04461	0,89530	0,54447

**maom**	**NAD-dependent malic enzyme, mitochondrial**	**Q99KE1**	**22**	**57%**	**0,86697**	**0,28223**	**0,79794**	**0,02883**
**mapk1**	**Mitogen-activated protein kinase 1**	**P63085**	**7**	**16%**	**0,82515**	**0,02731**	**0,66086**	**0,01112**
mk14	Mitogen-activated protein kinase 14	P47811	13	57%	1,76129	0,03954	1,74841	0,03047
mvp bas	Major vault protein	Q9EQK5	7	10%	1,74152	0,00741	1,30534	0,60262
mvp med	Major vault protein	Q9EQK5	14	18%	1,68139	0,00736	1,21356	0,63499
mvp ∑	Major vault protein	Q9EQK5			1,70938	0,00982	1,25629	0,61698
naa20	N-alpha-acetyltransferase 20	,P6160	5	35%	1,92416	0,00790	1,26842	0,07015
naga ac	N-acetylglucosamine-6-phosphate deacetylase	Q8JZV7	9	31%	1,26353	0,02255	0,93615	0,72583
ndus3	NADH dehydrogenase [ubiquinone] iron-sulfur protein 3, mitochondrial	Q9DCT2	5	23%	1,76446	0,00980	1,06141	0,71227
nit1	Nitrilase homolog 1	Q8VDK1	13	55%	0,53363	0,00972	0,82315	0,10447
nsf1c	NSFL1 cofactor p47	Q9CZ44	8	28%	0,75149	0,03603	1,23871	0,16724
odb2	Lipoamide acyltransferase component of branched-chain alpha-keto acid dehydrogenase complex, mitochondrial	P53395	18	42,00%	1,33892	0,03519	1,18731	0,24036
odo2	Dihydrolipoyllysine-residue succinyltransferase component of 2-oxoglutarate dehydrogenase complex, mitochondrial	Q9D2G2	9	24%	1,33185	0,01980	0,94383	0,79661
pcbp1	Poly(rC)-binding protein 1	P60335	14	75%	0,67657	0,04239	0,98541	0,92459
pdcd6	Programmed cell death protein 6	P12815	9	58%	1,32354	0,03975	1,05803	0,52904
pddc1	Parkinson disease 7 domain-containing protein 1	Q8BFQ8	3	19%	0,69947	0,20457	0,76998	0,01039
pdia1 ac	Protein disulfide-isomerase	P09103	20	42,00%	1,34229	0,13581	0,96016	0,84230
pdia1 med	Protein disulfide-isomerase	P09103	33	70,00%	1,60723	0,02123	1,02115	0,82547
pdia1 ∑	Protein disulfide-isomerase	P09103			1,46574	0,04547	0,93445	0,56375
pdxk	***Pyridoxal kinase***	***Q8K183***	***7***	***24%***	***1,46207***	***0,07857***	***0,58608***	***0,02273***
pgk1 ac	Phosphoglycerate kinase 1	P09411	20	62%	0,62818	0,02486	0,93025	0,41128
pgk1 bas	Phosphoglycerate kinase 1	P09411	25	69,00%	0,79014	0,04707	0,97776	0,75765
pgk1 sum	Phosphoglycerate kinase 1	P09411			0,69633	0,02968	0,95024	0,49578
pgls	6-phosphogluconolactonase	Q9CQ60	9	51%	1,48915	0,02618	0,94561	0,64183
phb	Prohibitin	P67778	12	53%	1,27375	0,00183	0,98638	0,78453

pipna	Phosphatidylinositol transfer protein alpha isoform	P53810	23	76%	0,70142	0,02782	0,87336	0,22829
**prps1**	**Ribose-phosphate pyrophosphokinase 1**	**,Q9D7G**	**10**	**39%**	**1,09710**	**0,56441**	**0,66283**	**0,01079**
prx3	Thioredoxin-dependent peroxide reductase, mitochondrial	P20108	3	11%	1,83406	0,02181	0,82291	0,37112
**prx6**	**Peroxiredoxin-6**	**O08709**	**17**	**80%**	**1,01587**	**0,82669**	**0,79438**	**0,00237**
psa1	Proteasome subunit alpha type-1	Q9R1P4	17	76%	1,34609	0,03449	0,86170	0,06202
**psb10**	**Proteasome subunit beta type-10**	**O35955**	**8**	**38%**	**1,45026**	**0,03400**	**0,64042**	**0,06162**
psb4 ac	Proteasome subunit beta type-4	P99026	3	16%	2,90441	0,03758	1,01946	0,93154
psb4 bas	Proteasome subunit beta type-4	P99026	8	49%	1,48629	0,14464	1,07429	0,32620
psb4 ∑	Proteasome subunit beta type-4	P99026			2,04997	0,07086	1,05250	0,49369
psmd2 ac	26S proteasome non-ATPase regulatory subunit 2	Q8VDM4	11	14%	1,56132	0,13670	1,05633	0,78149
psmd2 bas	26S proteasome non-ATPase regulatory subunit 2	Q8VDM4	13	16%	1,68826	0,03615	1,04733	0,79279
psmd2 ∑	26S proteasome non-ATPase regulatory subunit 2	Q8VDM4			1,63196	0,04585	1,05132	0,75403
psmd8	*26S proteasome non-ATPase regulatory subunit 8*	*Q9CX56*	*7*	*18%*	*1,26859*	*0,14314*	*0,69290*	*0,06247*
psmd14	26S proteasome non-ATPase regulatory subunit 14	O35593	5	18%	1,51073	0,04974	0,90995	0,61498
pth2	*Peptidyl-tRNA hydrolase 2, mitochondrial*	*Q8R2Y8*	*4*	*32%*	*1,58200*	*0,07167*	*1,02366*	*0,91501*
ran b3/1	Ran-binding protein 3	Q9CT10	8	20%	2,21322	0,04081	3,38495	0,06131
ranb3/2	Ran-binding protein 3	Q9CT10	10	24%	0,42471	0,05814	0,44310	0,00779
rap1a	Ras-related protein Rap-1A	P62835	3	21,00%	4,47577	0,03998	1,51363	0,35884
rbm8a	RNA-binding protein 8A	Q9CWZ3	5	51%	1,52292	0,07422	1,29914	0,19575
rm46	39S ribosomal protein L46, mitochondrial	Q9EQI8	5	22%	2,06179	0,01680	0,77167	0,15948
rssa	40S ribosomal protein SA	P14206	18	60%	1,40236	0,02817	0,97659	0,85186
**sarnp**	**SAP domain-containing ribonucleoprotein**	**Q9D1J3**	**5**	**28%**	**0,94784**	**0,44484**	**0,74430**	**0,00535**
sdcb1	Syntenin-1	O08992	3	26%	1,71727	0,02408	0,70053	0,05999
**sera**	**D-3-phosphoglycerate dehydrogenase**	**Q61753**	**3**	**7%**	**0,74962**	**0,05766**	**0,46038**	**0,02610**
sias	Sialic acid synthase OS=Homo sapiens	9	9	32%	0,52749	0,03753	0,91001	0,52460
snaa	Alpha-soluble NSF attachment protein	Q9DB05	13	49%	1,70178	0,03251	1,03238	0,86345

**snd1**	**Staphylococcal nuclease domain-containing protein 1**	**Q78PY7**	**17**	**28%**	**1,09108**	**0,66107**	**1,44869**	**0,00084**
**snx6 ac**	**Sorting nexin-6**	**Q6P8X1**	**9**	**25%**	**1,06397**	**0,79998**	**1,22126**	**0,03157**
**snx6 bas**	**Sorting nexin-6**	**Q6P8X1**	**18**	**44%**	**0,87703**	**0,41676**	**0,58176**	**0,00085**
snx6 ∑	Sorting nexin-6	Q6P8X1			0,96481	0,53074	0,88205	0,00774
**spb6 ac**	**Serpin B6**	**Q60854**	**13**	**39%**	**0,82831**	**0,11344**	**1,22955**	**0,02473**
spb6 bas	Serpin B6	Q60854	8	23%	1,56173	0,10652	1,07268	0,76891
spb6 med	Serpin B6	Q60854	9	26%	1,10774	0,59650	1,12246	0,48335
spb6 ∑	Serpin B6	Q60854			1,15245	0,42503	1,13442	0,39370
spee	Spermidine synthase	Q64674	2	7%	1,79253	0,04341	0,91716	0,44984
spre	Sepiapterin reductase	Q64105	13	63%	0,76223	0,03202	1,21136	0,39068
ssrd	Translocon-associated protein subunit delta	Q62186	4	31%	1,88099	0,01196	1,21136	0,39068
tmem11	Transmembrane protein 11, mitochondrial	Q8BK08	6	35%	1,50361	0,01978	1,20958	0,55435
tpm4	Tropomyosin alpha-4 chain	Q6IRU2	17	53%	0,38978	0,01036	0,50005	0,00116
twf2	Twinfilin-2	Q9Z0P5	10	38%	0,67903	0,02618	0,92104	0,64849
uba1	Ubiquitin-like modifier-activating enzyme 1	Q02053	25	34%	1,31969	0,02355	1,06249	0,79115
**ubql1h**	**Ubiquilin-1**	**Q8R317**	**2**	**3%**	**0,89348**	**0,16595**	**0,69245**	**0,01325**
ubql4	Ubiquilin-4 OS=Homo sapiens	Q9NRR5	2	3%	0,52405	0,02103	1,16653	0,71920
uchl5	Ubiquitin carboxyl-terminal hydrolase isozyme L5	Q9WUP7	7	21%	1,35660	0,00756	1,00065	0,99354
vata ac	V-type proton ATPase catalytic subunit A	P50516	6	12,80%	1,55768	0,03123	0,96678	0,86184
vata bas	V-type proton ATPase catalytic subunit A	P50516	23	42%	1,76854	0,01711	1,06525	0,74919
vata ∑	V-type proton ATPase catalytic subunit A	P50516			1,65676	0,01117	1,01305	0,94648
**vps29**	**Vacuolar protein sorting-associated protein 29**	**Q9QZ88**	**4**	**21%**	**0,99265**	**0,98566**	**0,67061**	**0,02975**
