# Supplementary material for: How Reversible Are the Effects of Fumed Silica on Macrophages? A Proteomics-Informed View
Source: Nanomaterials (Basel). 2020 Sep 29;10(10):1939. doi: 10.3390/nano10101939 (PMC7600894; doi:10.3390/nano10101939)
Supplement: Supplementary file 1 [file nanomaterials-10-01939-s001.zip › Table S2.pdf]

Supplementary table 2: Modulated pathways highlighted by the DAVID functional annotation clustering tool

**Annotation Cluster 1**	Enrichment Score: 3.7008879624375792					
Category	Term	Count	PValue	Genes	Fold Enrichment	FDR
GOTERM_MF_DIRECT	**GO:0098641~cadherin binding involved in cell-cell adhesion**	9	1,91E-05	P57776, P07356, P63101, P52480, Q9Z0P5, P24452, O08992, P06151, P60335	7,71	0,02467
GOTERM_CC_DIRECT	**GO:0005913~cell-cell adherens junction**	9	2,21E-05	P57776, P07356, P63101, P52480, Q9Z0P5, P24452, O08992, P06151, P60335	7,57	0,02725
GOTERM_CC_DIRECT	**GO:0042470~melanosome**	5	5,44E-04	P07356, P63101, P24452, O08992, P09103	13,15	0,66861
GOTERM_BP_DIRECT	**GO:0098609~cell-cell adhesion**	5	0,0068431	P57776, P63101, Q9Z0P5, P24452, P06151	6,55	9,42755

**Annotation Cluster 2**	Enrichment Score: 2.961292035066181					
Category	Term	Count	PValue	Genes	Fold Enrichment	FDR
UP_KEYWORDS	**Proteasome**	6	1,08E-06	P99026, O35955, O35593, Q9WUP7, Q9R1P4, Q8VDM4	32,40	0,00130
GOTERM_CC_DIRECT	**GO:0000502~proteasome complex**	6	4,60E-06	P99026, O35955, O35593, Q9WUP7, Q9R1P4, Q8VDM4	24,15	0,00567

UP_KEYWORDS	**Hydrolase**	17	6,58E-05	Q9CQ60, P56480, Q8VDK1, P99026, O70370, P97823, P50516, Q9Z1N5, O35955, O35593, Q9D819, Q9WUP7, Q9R1P4, Q8JZV7, Q99KK7, Q9Z0S1, Q9R0P3	3,12	0,07929
KEGG_PATHWAY	**mmu03050:Proteasome**	5	2,09E-04	P99026, O35955, O35593, Q9R1P4, Q8VDM4	16,43	0,24974
GOTERM_MF_DIRECT	GO:0016787~hydrolase activity	17	4,22E-04	Q9CQ60, P56480, Q8VDK1, P99026, O70370, P97823, P50516, Q9Z1N5, O35955, O35593, Q9D819, Q9WUP7, Q9R1P4, Q8JZV7, Q99KK7, Q9Z0S1, Q9R0P3	2,65	0,54354
GOTERM_MF_DIRECT	GO:0004175~endopeptidase activity	5	0,0011836	P99026, O35955, O35593, Q9R1P4, Q8VDM4	10,67	1,51804
GOTERM_BP_DIRECT	GO:0051603~proteolysis involved in cellular protein catabolic process	4	0,0015182	P99026, O70370, O35955, Q9R1P4	17,38	2,16721
UP_KEYWORDS	Threonine protease	3	0,0023256	P99026, O35955, Q9R1P4	41,24	2,76639
GOTERM_CC_DIRECT	GO:0005839~proteasome core complex	3	0,0024739	P99026, O35955, Q9R1P4	39,86	3,00761
INTERPRO	IPR001353:Proteasome, subunit alpha/beta	3	0,0025592	P99026, O35955, Q9R1P4	39,23	3,22737
GOTERM_MF_DIRECT	GO:0004298~threonine-type endopeptidase activity	3	0,0040178	P99026, O35955, Q9R1P4	31,17	5,06693
GOTERM_BP_DIRECT	GO:0002479~antigen processing and presentation of exogenous peptide antigen via MHC class I, TAP-dependent	3	0,0063285	P99026, O35955, Q9R1P4	24,77	8,74854

**Annotation Cluster 3**	Enrichment Score: 2.42781337900096					
Category	Term	Count	PValue	Genes	Fold Enrichment	FDR
KEGG_PATHWAY	**mmu01200:Carbon metabolism**	7	1,07E-04	Q9CQ60, Q9D2G2, P52480, P28271, Q9R0P3, P09411, P16858	8,93	0,12794
UP_KEYWORDS	**Glycolysis**	3	0,0058284	P52480, P09411, P16858	25,92	6,80087
GOTERM_BP_DIRECT	GO:0006096~glycolytic process	3	0,0090257	P52480, P09411, P16858	20,64	12,25596
KEGG_PATHWAY	mmu00010:Glycolysis / Gluconeogenesis	4	0,0093734	P52480, P06151, P09411, P16858	8,96	10,64844
KEGG_PATHWAY	mmu01230:Biosynthesis of amino acids	4	0,0137498	P52480, P28271, P09411, P16858	7,78	15,25506

**Annotation Cluster 4**	Enrichment Score: 2.290003037675952					
Category	Term	Count	PValue	Genes	Fold Enrichment	FDR
INTERPRO	IPR016040:NAD(P)-binding domain	7	8,10E-05	P50171, Q02053, Q91X52, P06151, Q64105, Q9CY64, P16858	9,66	0,10366
UP_KEYWORDS	NAD	6	3,32E-04	P50171, Q9DCT2, P06151, P24547, Q9CY64, P16858	9,91	0,39985
UP_KEYWORDS	**Oxidoreductase**	9	0,0011161	P20108, P50171, Q91X52, Q9DCT2, P06151, Q64105, P24547, Q9CY64, P16858	4,26	1,33652
GOTERM_MF_DIRECT	GO:0016491~oxidoreductase activity	8	0,012147	P20108, P50171, Q91X52, Q9DCT2, P06151, Q64105, P24547, Q9CY64	3,17	14,60231

**Annotation Cluster 5**	Enrichment Score: 2.221007657916773					
Category	Term	Count	PValue	Genes	Fold Enrichment	FDR
GOTERM_BP_DIRECT	**GO:0051016~barbed-end actin filament capping**	3	0,0018097	P47754, Q9Z0P5, P24452	46,44	2,57830
UP_KEYWORDS	**Actin-binding**	5	0,0093274	Q6IRU2, P40124, P47754, Q9Z0P5, P24452	6,00	10,67718
GOTERM_MF_DIRECT	GO:0003779~actin binding	6	0,0128705	Q9CQI3, Q6IRU2, P40124, P47754, Q9Z0P5, P24452	4,24	15,40666

**Annotation Cluster 6**	Enrichment Score: 1.7532557305124994					
Category	Term	Count	PValue	Genes	Fold Enrichment	FDR
UP_SEQ_FEATURE	metal ion-binding site:Magnesium 2	3	0,0140917	Q9D819, Q920E5, Q9Z0S1	16,37	16,94066
UP_SEQ_FEATURE	metal ion-binding site:Magnesium 1	3	0,0140917	Q9D819, Q920E5, Q9Z0S1	16,37	16,94066

**Annotation Cluster 7**	Enrichment Score: 1.554843385372					
Category	Term	Count	PValue	Genes	Fold Enrichment	FDR
INTERPRO	IPR013766:Thioredoxin domain	3	0,0090962	P20108, Q9CQM9, P09103	20,59	11,04033
GOTERM_BP_DIRECT	GO:0045454~cell redox homeostasis	3	0,0276759	P20108, Q9CQM9, P09103	11,43	33,28426
INTERPRO	IPR012336:Thioredoxin-like fold	3	0,086002	P20108, Q9CQM9, P09103	6,06	68,37724

**Annotation Cluster 8**	Enrichment Score: 1.3970844114629328					
Category	Term	Count	PValue	Genes	Fold Enrichment	FDR
UP_KEYWORDS	**Mitochondrion**	10	0,007354	P56480, Q9D2G2, P20108, P67778, P50171, Q8VDK1, Q02053, Q9DCT2, P53395, Q8BK08	2,86	8,50955
UP_KEYWORDS	Mitochondrion inner membrane	4	0,050366	P56480, P67778, Q9DCT2, Q8BK08	4,76	46,34913
GOTERM_CC_DIRECT	GO:0005743~mitochondrial inner membrane	4	0,1738136	P56480, P67778, Q9DCT2, Q8BK08	2,75	90,50128

**Annotation Cluster 9**	Enrichment Score: 1.345122814366088					
Category	Term	Count	PValue	Genes	Fold Enrichment	FDR
UP_KEYWORDS	**Mitochondrion**	10	0,007354	P56480, Q9D2G2, P20108, P67778, P50171, Q8VDK1, Q02053, Q9DCT2, P53395, Q8BK08	2,86	8,50955
UP_KEYWORDS	Transit peptide	5	0,0853392	P56480, Q9D2G2, P20108, Q9DCT2, P53395	2,96	65,86303
UP_SEQ_FEATURE	transit peptide:Mitochondrion	5	0,1468782	P56480, Q9D2G2, P20108, Q9DCT2, P53395	2,42	87,47729
