# Supplementary material for: How Reversible Are the Effects of Fumed Silica on Macrophages? A Proteomics-Informed View
Source: Nanomaterials (Basel). 2020 Sep 29;10(10):1939. doi: 10.3390/nano10101939 (PMC7600894; doi:10.3390/nano10101939)
Supplement: Supplementary file 1 [file nanomaterials-10-01939-s001.zip › Table S3.pdf]

Supplementary Table 3: comparison of proteins showing a significant change after exposure to pyrolytic or colloidal silica [10]

I: increase in treated compared to control ; D: decrease in treated compared to control ; NC: no significant change when present : ac=acidic form; bas=basic form : med=median form

code	name	accession	pyrolytic silica	colloidal silica
14-3-3 eps	14-3-3 protein epsilon	P62259	NC	D
14-3-3 zeta	14-3-3 protein zeta/delta	P63101	D	NC
aars ac	Alanine--tRNA ligase, cytoplasmic	Q8BGQ7	I	NC
aars bas	Alanine--tRNA ligase, cytoplasmic	Q8BGQ7	I	NC
acadl	Long-chain specific acyl-CoA dehydrogenase	Q8BFR5	NC	I
acoc	Cytoplasmic aconitate hydratase	P28271	D	NC
adk	adenosine kinase	P55264	NC	D
amrp	Alpha-2-macroglobulin receptor-associated protein	P55302	D	I
anxa1	Annexin A1	P10107	NC	I
anxa2	Annexin A2	P07356	D	NC
anxa5 ac	Annexin A5	P48036	D	NC
anxa5 bas	Annexin A5	P48036	D	NC
ap3m1	AP-3 complex subunit mu-1	Q9JKC8	I	NC
arl3	ADP-ribosylation factor-like protein 3	Q9WUL7	I	D
atpb ac	ATP synthase subunit beta, mitochondrial	P56480	I	NC
atpb bas	ATP synthase subunit beta, mitochondrial	P56480	I	NC
bid ac.	BH3-interacting domain death agonist	P70444	NC	D
blvrb	Biliverdin reductase B	Q923D2	NC	I
bpnt1	3'(2'),5'-bisphosphate nucleotidase 1	Q9Z0S1	I	I
brcc3	Lys63-deubiquitinase brcc36	P46737	NC	D
bt3L4	Transcription factor BTF3 homolog 4	Q9CQH7	NC	D
bvra ac	Biliverdin reductase A	Q9CY64	D	D
bvra bas	Biliverdin reductase A	Q9CY64	D	NC
cab39 ac	Calcium-binding protein 39	Q06138	NC	D
cah13	Carbonic anhydrase 13	Q9D6N1	D	NC
cap1	Adenylyl cyclase-associated protein 1	P40124	D	NC
capg	Macrophage-capping protein	P24452	D	NC
capg ac	Macrophage-capping protein	P24452	NC	NC
Casp 3 bas	Caspase-3	P70677	NC	D
casp1	Caspase-1	P29452	NC	D
catS	Cathepsin S	O70370	D	NC
catZ	Cathepsin Z	Q9WUU7	NC	D
caza2 ac	F-actin-capping protein subunit alpha-2	P47754	I	D
cdc42	Cell division control protein 42 homolog	P60766	NC	D
cdk6	Cyclin-dependent kinase 6	Q64261	D	NC
clic4 ac	Chloride intracellular channel protein 4	Q9QYB1	NC	I
clic4 bas	Chloride intracellular channel protein 4	Q9QYB1	NC	I
cof bas	Cofilin-1	P18760	D	NC
cof ac	Cofilin-1, monophosphorylated form	P18760	NC	D
coq9	Ubiquinone biosynthesis protein COQ9, mitochondrial	Q8K1Z0	NC	I
cpped	Serine/threonine-protein phosphatase CPPED1	Q8BFS6	NC	D

cpsn1 ac	Calpain small subunit 1	O88456	D	NC
cpsn1 bas	Calpain small subunit 1	O88456	NC	NC
cryl1	Lambda-crystallin homolog	Q9CQT1	NC	D
ctbp1	C-terminal-binding protein 1	O88712	NC	D
dcup	Uroporphyrinogen decarboxylase	P70697	D	NC
dcxr	L-xylulose reductase	Q91X52	D	NC
ddx39b	Spliceosome RNA helicase Ddx39b	Q9Z1N5	I	NC
dhb8	Estradiol 17-beta-dehydrogenase 8	P50171	I	NC
dj1 ac	Protein deglycase DJ-1	Q99LX0	D	NC
dj1 bas	Protein deglycase DJ-1	Q99LX0	I	NC
dnjc9	DnaJ homolog subfamily C member 9	Q91WN1	NC	D
dnph1	2'-deoxynucleoside 5'-phosphate N-hydrolase 1	Q80VJ3	NC	D
dpp3 ac	Dipeptidyl peptidase 3	Q99KK7	D	NC
dpp3 bas	Dipeptidyl peptidase 3	Q99KK7	I	NC
ef1d	Elongation factor 1-delta	P57776	I	NC
eftu	Elongation factor Tu, mitochondrial	Q8BFR5	NC	I
esd	S-formylglutathione hydrolase	Q9R0P3	I	NC
fa49b	Protein FAM49B	Q921M7	NC	D
fkbp4 ac	Peptidyl-prolyl cis-trans isomerase FKBP4	P30416	NC	I
fkbp4 bas	Peptidyl-prolyl cis-trans isomerase FKBP4	P30416	NC	NC
fkbp4 med	Peptidyl-prolyl cis-trans isomerase FKBP4	P30416	I	I
fpps ac	Farnesyl pyrophosphate synthase	Q920E5	NC	NC
fpps bas	Farnesyl pyrophosphate synthase	Q920E5	I	I
frih	Ferritin heavy chain	P09528	NC	I
galK ac	Galactose kinase	Q9R0N0	NC	D
galK bas	Galactose kinase	Q9R0N0	NC	NC
gapdh	Glyceraldehyde-3-phosphate dehydrogenase	P16858	D	NC
gbb1	Guanine nucleotide-binding protein G(I)/G(S)/G(T) subunit beta-1	P62874	I	I
gbb2	Guanine nucleotide-binding protein G(I)/G(S)/G(T) subunit beta-2	P62880	NC	I
glrx3 ac	Glutaredoxin-3	Q9CQM9	NC	NC
glrx3 bas	Glutaredoxin-3	Q9CQM9	I	NC
gmfb	Glia maturation factor beta	Q9CQI3	D	NC
grp75	Stress-70 protein, mitochondrial	O35501	I	NC
hmgCL	Hydroxymethylglutaryl-CoA lyase, mitochondrial	P38060	NC	D
hnrl2	Heterogeneous nuclear ribonucleoprotein U-like protein 2	Q00PI9	D	NC
htra2	Serine protease HTRA2, mitochondrial	Q9JIY5	NC	I
hyou1 ac	Hypoxia up-regulated protein 1	Q9JKR6	NC	I
hyou1 bas	Hypoxia up-regulated protein 1	Q9JKR6	NC	I
hyou1 med	Hypoxia up-regulated protein 1	Q9JKR6	NC	I
idhc ac	Isocitrate dehydrogenase, cytoplasmic	O88844	NC	D
idhc bas	Isocitrate dehydrogenase, cytoplasmic	O88844	NC	NC
if2b	Translation initiation factor 2 subunit 2	Q99L45	NC	I
iF3M	Eukaryotic translation initiation factor 3 subunit M	Q99JX4	I	NC
ima7	Importin subunit alpha-7	O35345	I	NC
in35	Interferon-induced 35 kDa protein homolog	Q9D8C4	NC	D
ipyr	Inorganic pyrophosphatase	Q9D819	I	NC

kcy	UMP-CMP kinase	Q9DBP5	D	NC
kpym ac	Pyruvate kinase PKM	P52480	D	NC
kpym bas	Pyruvate kinase PKM	P52480	D	NC
ldha	L-lactate dehydrogenase A chain	P06151	D	NC
lgul	Lactoylglutathione lyase	Q9CPU0	NC	D
lhpp	Phospholysine phosphohistidine inorganic pyrophosp	Q9D7I5	NC	I
lsp1 ac	Lymphocyte-specific protein 1	P19973	NC	NC
lsp1 bas	Lymphocyte-specific protein 1	P19973	NC	D
lypa1	Acyl-protein thioesterase 1	P97823	I	NC
mat2b	Methionine adenosyltransferase 2 subunit beta	Q99LB6	NC	D
mk14	Mitogen-activated protein kinase 14	P47811	I	I
mtna	Methylthioribose-1-phosphate isomerase	Q9CQT1	NC	I
mtx2	Metaxin-2	O88441	NC	D
mvp bas	Major vault protein	Q9EQK5	I	NC
mvp med	Major vault protein	Q9EQK5	I	NC
myd88	Myeloid differentiation primary response protein My	P22366	NC	D
naa20	N-alpha-acetyltransferase 20	,P6160	I	NC
naga ac	N-acetylglucosamine-6-phosphate deacetylase	Q8JZV7	I	NC
ndus3	NADH dehydrogenase [ubiquinone] iron-sulfur protein 3, mitochondrial	Q9DCT2	I	NC
nit1	Nitrilase homolog 1	Q8VDK1	D	NC
nmrl1	NmrA-like family domain-containing protein 1	Q8K2T1	NC	D
nsf1c	NSFL1 cofactor p47	Q9CZ44	D	D
odb2	Lipoamide acyltransferase component of branched-chain alpha-keto acid dehydrogenase complex, mitochondrial	P53395	I	NC
odo2	Dihydrolipoyllysine-residue succinyltransferase component of 2-oxoglutarate dehydrogenase complex, mitochondrial	Q9D2G2	I	NC
odp2 ac	Dihydrolipoyllysine-residue acetyltransferase compo	Q8BMF4	NC	I
osgep	Probable tRNA N6-adenosine threonylcarbamoyltransferase	Q8BWU5	NC	D
pcbp1	Poly(rC)-binding protein 1	P60335	D	NC
pcna ac	Proliferating cell nuclear antigen	P17918	NC	D
pcna bas	Proliferating cell nuclear antigen	P17918	NC	NC
pdcd6	Programmed cell death protein 6	P12815	I	NC
pddc1	Parkinson disease 7 domain-containing protein 1	Q8BFQ8	D	D
pde6d	Retinal rod rhodopsin-sensitive cGMP 3',5'-cyclic ph	O55057	NC	I
pdia1 ac	Protein disulfide-isomerase	P09103	NC	NC
pdia1 med	Protein disulfide-isomerase	P09103	I	NC
pfkal	6-phosphofructokinase, liver type	P12382	NC	I
pgk1 ac	Phosphoglycerate kinase 1	P09411	D	NC
pgk1 bas	Phosphoglycerate kinase 1	P09411	D	NC
pgls	6-phosphogluconolactonase	Q9CQ60	I	NC
phb	Prohibitin	P67778	I	I
pihd1	PIH1 domain-containing protein 1	Q9WTM5	NC	D
pipna	Phosphatidylinositol transfer protein alpha isoform	P53810	D	NC
pipnb	Phosphatidylinositol transfer protein beta isoform	P53811	NC	D
ppac	Low molecular weight phosphotyrosine protein phos	Q9D358	NC	D
ppie	Peptidyl-prolyl cis-trans isomerase E	Q9QZH3	NC	I

ppp1ca	Serine/threonine-protein phosphatase PP1-alpha catalytic subunit	P62137	NC	D
prdx1ox	Peroxiredoxin 1, oxidized form	P35700	NC	I
prps1 a	Ribose-phosphate pyrophosphokinase 1	Q9D7G0	NC	I
prps1 b	Ribose-phosphate pyrophosphokinase 1	Q9D7G0	NC	NC
prs7	Proteasome regulatory subunit 7	P46471	NC	I
prx3	Thioredoxin-dependent peroxide reductase, mitochondrial	P20108	I	NC
psa1	Proteasome subunit alpha type-1	Q9R1P4	I	NC
psb3 ac.	Proteasome beta 3 subunit	Q9R1P1	NC	D
psb3 bas	Proteasome beta 3 subunit	Q9R1P1	NC	I
psb4 ac	Proteasome subunit beta type-4	P99026	I	NC
psb4 bas	Proteasome subunit beta type-4	P99026	I	I
psd10	Proteasome regulatory subunit 10	Q9Z2X2	NC	I
psd13	Proteasome regulatory subunit 13	Q9UNM6	NC	I
psmd14	26S proteasome non-ATPase regulatory subunit 14	O35593	I	NC
psmd2 ac	26S proteasome non-ATPase regulatory subunit 2	Q8VDM4	I	NC
psmd2 bas	26S proteasome non-ATPase regulatory subunit 2	Q8VDM4	I	NC
psmd8	Proteasome regulatory subunit 8	Q9CX56	NC	D
psme2	Proteasome activator complex subunit 2	P97372	NC	I
pth2	Peptidyl-tRNA hydrolase 2, mitochondrial	Q8R2Y8	I	NC
ptpn6 ac	Tyrosine-protein phosphatase non-receptor type 6	P29351	NC	D
ptpn6 bas	Tyrosine-protein phosphatase non-receptor type 6	P29351	NC	NC
rab14	Ras-related protein Rab-14	Q91V41	NC	D
ran b3/1	Ran-binding protein 3	Q9CT10	I	NC
ranb3/2	Ran-binding protein 3	Q9CT10	D	NC
rap1a	Ras-related protein Rap-1A	P62835	I	NC
rbm8a	RNA-binding protein 8A	Q9CWZ3	I	NC
rfa2	Replication protein A 32 kDa subunit	Q62193	NC	D
rfc2	Replication factor C subunit 2	P35250	NC	D
rhoA	Transforming protein RhoA	Q9QUI0	NC	D
rm46	39S ribosomal protein L46, mitochondrial	Q9EQI8	I	NC
rras	Ras-related protein R-Ras	P10833	NC	D
rssa	40S ribosomal protein SA	P14206	I	NC
sae1 ac	SUMO-activating enzyme subunit 1	Q9R1T2	NC	D
sae1 bas	SUMO-activating enzyme subunit 1	Q9R1T2	NC	D
sarnp	SAP domain-containing ribonucleoprotein	Q9D1J3	NC	D
sdcb1	Syntenin-1	O08992	I	NC
sias	Sialic acid synthase OS=Homo sapiens	D	D	NC
snaa	Alpha-soluble NSF attachment protein	Q9DB05	I	NC
snap23	Synaptosomal-associated protein 23	O09044	NC	D
snx6 ac.	Sorting nexin 6	Q6P8X1	NC	D
spb6 bas	Serpin B6	Q60854	I	NC
spb6 med	Serpin B6	Q60854	NC	NC
spee	Spermidine synthase	Q64674	I	NC
spre	Sepiapterin reductase	Q64105	D	D
ssrd	Translocon-associated protein subunit delta	Q62186	I	I
strap ac	Serine-threonine kinase receptor-associated protein	Q9Z1Z2	NC	D

strap bas	Serine-threonine kinase receptor-associated protein	Q9Z1Z2	NC	NC
sucb2 ac	Succinate-CoA ligase [GDP-forming] subunit beta, mitochondrial	Q9Z2I8	NC	I
sucb2 bas	Succinate-CoA ligase [GDP-forming] subunit beta, mitochondrial	Q9Z2I8	NC	I
tatd3	Putative deoxyribonuclease TATDN3	Q3U1C6	NC	D
tcpz	T-complex protein 1 subunit zeta	P80317	NC	D
thtm	3-mercaptopyruvate sulfurtransferase	Q99J99	NC	I
tmem11	Transmembrane protein 11, mitochondrial	Q8BK08	I	NC
tpm4	Tropomyosin alpha-4 chain	Q6IRU2	D	NC
tsnax	Translin-associated protein X	Q9QZE7	NC	D
twf1	Twinfilin-1	Q91YR1	NC	I
twf2	Twinfilin-2	Q9Z0P5	D	D
txd12	Thioredoxin domain-containing protein 12	Q9CQU0	NC	D
uba1	Ubiquitin-like modifier-activating enzyme 1	Q02053	I	NC
ube2n	Ubiquitin conjugating enzyme 2N	P61088	NC	D
ubql4	Ubiquilin-4 OS=Homo sapiens	Q9NRR5	D	NC
uchl5	Ubiquitin carboxyl-terminal hydrolase isozyme L5	Q9WUP7	I	NC
vata ac	V-type proton ATPase catalytic subunit A	P50516	I	NC
vata bas	V-type proton ATPase catalytic subunit A	P50516	I	NC
vdac2	Voltage-dependent anion-selective channel protein 2	Q60930	NC	I
vps25	Vacuolar protein-sorting-associated protein 25	Q9CQ80	NC	D
