# Supplementary material for: How Reversible Are the Effects of Fumed Silica on Macrophages? A Proteomics-Informed View
Source: Nanomaterials (Basel). 2020 Sep 29;10(10):1939. doi: 10.3390/nano10101939 (PMC7600894; doi:10.3390/nano10101939)
Supplement: Supplementary file 1 [file nanomaterials-10-01939-s001.zip › Table S4.pdf]

Supplementary table 4: Modulated pathways highlighted by the DAVID functional annotation clustering tool, for cells treated with colloidal silica [10]

**Annotation Cluster 1**	Enrichment Score: 4,18144249257887					
Category	Term	Count	PValue	Genes	Fold Enrichment	FDR
GOTERM_CC_DIRECT	**GO:0000502~proteasome complex**	6	1,09E-05	P97372, Q9CX56, P99026, P46471, Q9R1P1, Q9Z2X2	20,31	0,00044
UP_KEYWORDS	**Proteasome**	5	6,66E-05	P97372, Q9CX56, P99026, P46471, Q9R1P1	22,75	0,00266
KEGG_PATHWAY	**mmu03050:Proteasome**	5	3,93E-04	P97372, Q9CX56, P99026, P46471, Q9R1P1	14,01	0,02710

**Annotation Cluster 2**	Enrichment Score: 2,284642433915229					
Category	Term	Count	PValue	Genes	Fold Enrichment	FDR
INTERPRO	**IPR002108:Actin-binding, cofilin/tropomyosin type**	3	7,77E-04	Q9Z0P5, P18760, Q91YR1	70,21	0,19105
GOTERM_BP_DIRECT	**GO:0051016~barbed-end actin filament capping**	3	0,00228343	Q9Z0P5, P47754, Q91YR1	41,35	0,96008
UP_KEYWORDS	**Actin-binding**	4	0,07486874	Q9Z0P5, P47754, P18760, Q91YR1	4,04	0,39930
GOTERM_MF_DIRECT	**GO:0003779~actin binding**	5	0,074912681	Q9Z0P5, P47754, P18760, Q91YR1, P19973	3,11	0,75848

**Annotation Cluster 3**	Enrichment Score: 2,2736727703379787					
Category	Term	Count	PValue	Genes	Fold Enrichment	FDR
UP_KEYWORDS	NADP	6	6,03E-04	Q923D2, Q9CY64, O88844, Q99LB6, Q8K2T1, Q64105	8,74	0,01609
UP_SEQ_FEATURE	binding site:NADP	3	0,006581131	O88844, Q8K2T1, Q64105	24,29	0,58387
UP_SEQ_FEATURE	nucleotide phosphate-binding region:NADP	3	0,038024095	O88844, Q8K2T1, Q64105	9,64	0,88596

**Annotation Cluster 4**	Enrichment Score: 1,9799880012073776					
Category	Term					
UP_KEYWORDS	**Tricarboxylic acid cycle**	Count	PValue	Genes	Fold Enrichment	FDR
KEGG_PATHWAY	mmu01130:Biosynthesis of antibiotics	3	0,004213574	O88844, Q9Z2I8, Q8BMF4	30,58	0,05618

GOTERM_BP_DIRECT	GO:0006099~tricarboxylic acid cycle	7	0,006195389	P12382, Q920E5, O88844, Q9Z0S1, Q9Z2I8, Q8BMF4, Q9D7G0	4,12	0,19892
KEGG_PATHWAY	**mmu01200:Carbon metabolism**	3	0,007439951	O88844, Q9Z2I8, Q8BMF4	22,81	0,96008
KEGG_PATHWAY	mmu01230:Biosynthesis of amino acids	5	0,012523794	P12382, O88844, Q9Z2I8, Q8BMF4, Q9D7G0	5,43	0,24690
KEGG_PATHWAY	mmu00020:Citrate cycle (TCA cycle)	4	0,021214743	P12382, O88844, Q99LB6, Q9D7G0	6,64	0,34820
		3	0,02555107	O88844, Q9Z2I8, Q8BMF4	11,82	0,35260
**Annotation Cluster 5**	Enrichment Score: 1,8522219849064838					
Category	Term					
UP_KEYWORDS	NADP	Count	PValue	Genes	Fold Enrichment	FDR
UP_KEYWORDS	**Oxidoreductase**	6	6,03E-04	Q923D2, Q9CY64, O88844, Q99LB6, Q8K2T1, Q64105	8,74	0,01609
GOTERM_MF_DIRECT	GO:0016491~oxidoreductase activity	8	0,012119744	P09528, Q923D2, Q9CY64, O88844, Q9CQU0, P62259, P35700, Q64105	3,19	0,12928
GOTERM_BP_DIRECT	GO:0055114~oxidation-reduction process	7	0,064679029	P09528, Q923D2, Q9CY64, O88844, Q9CQU0, P35700, Q64105	2,44	0,70853
		7	0,082458015	P09528, Q923D2, Q9CY64, O88844, Q9CQU0, P35700, Q64105	2,28	1,00000
**Annotation Cluster 6**	Enrichment Score: 1,8148662468770735					
Category	Term					
UP_KEYWORDS	**Mitochondrion**	Count	PValue	Genes	Fold Enrichment	FDR
UP_KEYWORDS	Transit peptide	13	7,77E-04	Q8BFR5, Q60930, P30416, Q9Z2I8, P70444, Q8BMF4, Q99J99, P67778, Q8K1Z0, P38060, O88441, Q9JIY5, Q9QYB1	3,13	0,01777
UP_SEQ_FEATURE	transit peptide:Mitochondrion	6	0,048392576	Q8BFR5, Q8K1Z0, P38060, Q9Z2I8, Q8BMF4, Q9JIY5	3,00	0,33173
		6	0,095493551	Q8BFR5, Q8K1Z0, P38060, Q9Z2I8, Q8BMF4, Q9JIY5	2,45	1,00000
**Annotation Cluster 7**	Enrichment Score: 1,7170923275646146					

Category	Term					
GOTERM_MF_DIRECT	**GO:0098641~cadherin binding involved in cell-cell adhesion**	Count	PValue	Genes	Fold Enrichment	FDR
GOTERM_CC_DIRECT	**GO:0005913~cell-cell adherens junction**	6	0,010125714	P10107, Q9Z0P5, O88844, Q91YR1, P62259, P35700	4,52	0,22185
GOTERM_BP_DIRECT	**GO:0098609~cell-cell adhesion**	6	0,013145635	P10107, Q9Z0P5, O88844, Q91YR1, P62259, P35700	4,24	0,20527
		4	0,053029287	Q9Z0P5, O88844, Q91YR1, P35700	4,67	1,00000
**Annotation Cluster 8**	Enrichment Score: 1,691469256512831					
Category	Term					
UP_KEYWORDS	Magnesium	Count	PValue	Genes	Fold Enrichment	FDR
UP_KEYWORDS	Metal-binding	9	9,45E-04	P12382, Q9QZE7, Q920E5, Q9WUL7, O88844, P55264, Q9Z0S1, Q9D7I5, Q9D7G0	4,40	0,01891
GOTERM_MF_DIRECT	GO:0046872~metal ion binding	20	0,060363647	P09528, Q8BFS6, Q9QZE7, Q920E5, P10107, P55264, Q9CY64, Q99L45, Q9CPU0, P46737, Q8BWU5, P12382, Q9WUL7, O88844, Q9Z0S1, P38060, Q9D7I5, P62137, Q9D7G0, Q3U1C6	1,50	0,35771
		21	0,147647788	P09528, Q8BFS6, Q9QZE7, Q920E5, P10107, P55264, Q9CY64, Q99L45, Q9Z2I8, Q9CPU0, P46737, Q8BWU5, P12382, Q9WUL7, O88844, Q9Z0S1, P38060, Q9D7I5, P62137, Q9D7G0, Q3U1C6	1,32	0,97571
